# Influence of Basal Medium and Organic Additives on In Vitro Germination and Plant Growth of Endangered Orchid *Gastrochilus fuscopunctatus*

**DOI:** 10.3390/plants14203133

**Published:** 2025-10-11

**Authors:** Jung Eun Hwang, Hyeong Bin Park, Jae-Hwa Tho, Myojin Kim, Hwan Joon Park, Seongjun Kim, Chang Woo Lee, Young-Joong Kim

**Affiliations:** 1 Research Center for Endangered Species, National Institute of Ecology, Yeongyang 36531, Republic of Korea; phb1274@nie.re.kr (H.B.P.); norgak@nie.re.kr (J.-H.T.); kmjadspopli@nie.re.kr (M.K.); rhg9281@nie.re.kr (H.J.P.); dao1229@nie.re.kr (S.K.); jacky903@nie.re.kr (C.W.L.); yjkim@nie.re.kr (Y.-J.K.); 2 Department of Environmental Horticulture, University of Seoul, Seoul 02504, Republic of Korea

**Keywords:** *Gastrochilus fuscopunctatus*, endangered orchid, asymbiotic seed germination, Hyponex medium, banana homogenate, in vitro propagation

## Abstract

*Gastrochilus fuscopunctatus* is a rare epiphytic orchid in Korea threatened by habitat loss and illegal collection. This study aimed to establish an efficient in vitro propagation system by evaluating asymbiotic germination and seedling growth. Mature seeds germinated on both Hyponex (Hy) and Orchid Seed Sowing Medium (OSM), but protocorm development was more active on Hy, regardless of 1 µM NAA supplementation. For seedling culture, Murashige and Skoog (MS), Hy, and Orchid Maintenance Medium (OM) were tested with apple homogenate (AH), banana homogenate (BH), and coconut water (CW). At 7 months, Hy supported the greatest biomass and root formation, while Hy + BH produced the highest values (0.066 g fresh weight, 1.3 cm root length). Although BH improved growth on MS and OM, the effects were less pronounced. At 14 months, Hy + BH again yielded superior results (1.93 g fresh weight, 5.3 cm root length, 17.2 leaves), clearly outperforming all other combinations. AH and CW showed inconsistent or limited effects across media, indicating strong medium–additive interactions. These findings demonstrate that Hy + BH is the most effective combination for both early and prolonged growth of *G. fuscopunctatus*, providing a practical framework for ex situ conservation and reintroduction.

## 1. Introduction

The orchid family (Orchidaceae) is widely recognized as one of the most diverse and ecologically significant groups of angiosperms, comprising approximately 736 genera and more than 28,000 species worldwide [1,2]. Despite this impressive richness, a considerable number of species—approximately 1300—are currently included in the International Union for Conservation of Nature (IUCN) Red List, with many classified as endangered or critically endangered [3]. The major threats contributing to the decline of orchid populations include habitat destruction caused by urbanization and deforestation, unsustainable resource management, environmental pollution, and excessive harvesting for horticultural and medicinal purposes [4,5].

Another key factor that makes orchids particularly vulnerable is their unique reproductive biology. Orchid seeds are characteristically minute, lack endosperm, and are physiologically incapable of utilizing stored lipids. As a result, they depend almost entirely on symbiotic associations with mycorrhizal fungi for germination in nature. In the absence of suitable fungal partners, germination rates remain extremely low, and even when germination occurs, seedling growth is slow and highly inefficient [6,7]. This biological limitation presents a substantial challenge for the recovery of wild populations, particularly for rare and endangered taxa.

Conventional propagation methods such as clump division and the back-bulb culture have long been used for horticultural purposes. However, these approaches are inherently slow, labor-intensive, and yield only a limited number of propagules over extended periods, rendering them unsuitable for large-scale conservation programs. In contrast, modern in vitro tissue culture techniques offer a powerful alternative, as they enable rapid and large-scale propagation, enhance germination and seedling growth, and maintain genetic fidelity. For rare and endangered orchid species, tissue culture is now widely regarded as an indispensable strategy for both ex situ conservation and ecological restoration [8]. Within the scope of tissue culture, asymbiotic seed germination has emerged as a particularly valuable method because it bypasses the dependency on mycorrhizal fungi and allows seeds to germinate under controlled laboratory conditions. Since in vitro germination experiments are cheap and fast methods for species that have difficulty in germinating, a healthier and higher percentage of germination can be achieved in the early germination stages of plants by creating different fertilizers and nutrient media [9,10]. Nevertheless, the efficiency of this approach is strongly influenced by several biological and technical factors, including the developmental stage of the embryo, capsule maturity, and especially the formulation of the basal medium and the presence of plant growth regulators or supplementary additives [11,12]. Previous studies have demonstrated that optimizing the nutritional composition of the medium, and the judicious use of growth regulators such as auxins and cytokinins, as well as additives like activated charcoal or peptone, can significantly enhance protocorm development and seedling establishment in numerous orchid species [13,14].

In addition to inorganic nutrient formulations, organic supplements have attracted considerable attention for their role in promoting in vitro regeneration, multiplication, and seedling vigor [15]. Because the physiological requirements of orchids vary greatly among taxa, the optimal type and concentration of these supplements must be carefully determined on a species-specific basis [14,16]. A broad range of natural additives—including apple juice, homogenized banana and potato, coconut water, tomato juice, honey, corn extract, yeast extract, and papaya extract—have been reported to stimulate cell division, differentiation, and morphogenesis in orchids [15,17]. Such organic additives not only improve propagation efficiency but also enhance the overall quality and vigor of regenerated plants, making them valuable tools for orchid biotechnology and conservation programs [16,18].

**Figure 1:**
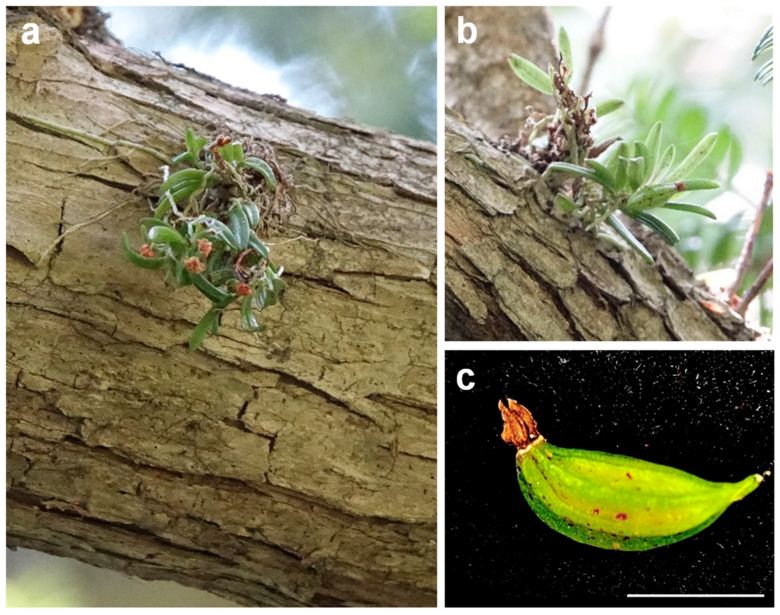
Morphology of *G. fuscopunctatus* in its natural habitat. (**a**) Flowering plant attached to bark of Torreya nucifera, showing clustered shoots with opposite leaves and characteristic purple-spotted foliage. (**b**) Immature capsule developing at leaf axil. (**c**) Fully mature capsule containing numerous dust-like seeds. Scale bar = 1 cm.

*Gastrochilus fuscopunctatus*, commonly known as Geumjaran in Korea, is a diminutive epiphytic orchid typically found growing on the bark of *Torreya nucifera* and *Pinus* species (Figure 1a,b). The entire plant rarely exceeds 10 cm in length, with a short stem of about 5 cm that bears multiple nodes, each producing characteristic white aerial roots that firmly attach to tree bark. The elliptic leaves, approximately 1 cm in length, are arranged alternately in two rows along the stem and exhibit distinctive purple spotting (Figure 1a,b). From April to May, small inflorescences, roughly 1 cm long, emerge from the leaf axils and bear one to four pale yellow-green flowers densely covered with purple spots, with a prominent labellum and well-developed floral structures (Figure 1a). Mature capsules are green and contain numerous dust-like seeds adapted for wind dispersal (Figure 1c).

Geographically, this species is distributed across China, Taiwan, and Japan, but in Korea, its range is highly restricted, confined only to Jeju Island and a few offshore islets in Namhae-gun, Gyeongsangnam-do [19]. Due to overcollection, limited distribution, and rapid habitat degradation, natural populations have undergone a sharp decline, prompting their classification as an Endangered Wildlife Category I species in Korea [20]. This species was selected for study because of its extremely limited natural populations and its ecological importance as part of the unique epiphytic orchid flora of Korea. In addition, its horticultural potential and value for biodiversity conservation make the development of efficient propagation methods highly relevant. Although Kang et al. [21] investigated the influence of plant growth regulators on the in vitro culture of *G. fuscopunctatus*, the potential effects of organic supplements on its germination and seedling development remain largely unexplored.

Therefore, the present study was undertaken to systematically evaluate the effects of different organic supplements on the asymbiotic seed germination and early seedling growth of *G. fuscopunctatus*. By identifying the most effective additives and elucidating their role in promoting propagation efficiency, this research provides essential insights for the development of optimized tissue culture protocols. Ultimately, the findings are expected to contribute to both the ex situ conservation and the restoration of wild populations of this endangered orchid.

## 2. Results and Discussion

### 2.1. Effects of Basal Medium Composition on Asymbiotic Germination and Early Protocorm Development

**Figure 2:**
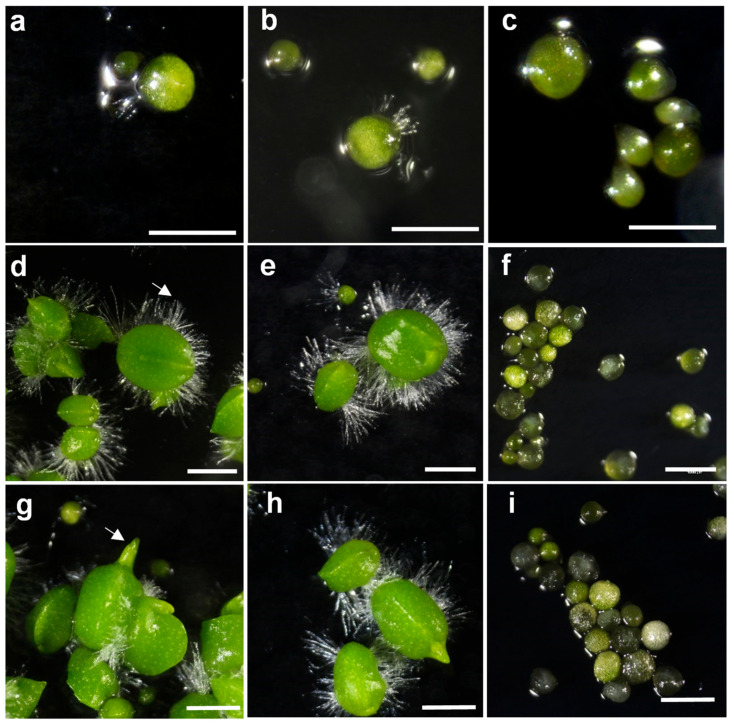
Asymbiotic seed germination and protocorm development of *G. fuscopuncta* on different culture media. (**a**,**d**,**g**) Hyponex medium; (**b**,**e**,**h**) Hyponex medium supplemented with 1 µM NAA; (**c**,**f**,**i**) Orchid Seed Sowing Medium (OSM). Images captured at (**a**–**c**) 3 weeks, (**d**–**f**) 6 weeks, and (**g**–**i**) 8 weeks after sowing. White arrow in (**d**) indicates rhizoids, and arrows in (**g**) indicate developing shoot primordia. Scale bar = 1 mm.

Seed germination and protocorm development in orchids are known to be strongly influenced by the composition of the basal medium and the presence of plant growth regulators such as NAA [22]. In the present study, we evaluated the effects of Hyponex (Hy) and Orchid Seed Sowing Medium (OSM) on the germination and protocorm development of *G. fuscopunctatus*. To further assess the role of plant growth regulators, NAA was supplemented in selected treatments for comparison. Three weeks after sowing, seeds germinated and protocorm development progressed normally to stage 3 in all three tested media—Hy, Hy supplemented with 1 µM NAA, and OSM (Figure 2a–c). At this stage, the rupture of the testa and exposure of the embryo were observed, with no apparent morphological differences among the treatments.

By six weeks, however, distinct differences emerged among the media. In both Hyponex and Hyponex + NAA media, well-defined protomeristems had formed, accompanied by the development of numerous rhizoids on the protocorm surface (Figure 2d,e). The protocorms in these treatments exhibited noticeable enlargement and active cell division, suggesting a progression toward subsequent shoot and root differentiation. In contrast, protocorms cultured on SGM remained arrested at stage 3 with little or no morphological advancement (Figure 2f).

After eight weeks, the developmental disparity between the treatments became even more pronounced. Protocorms on Hyponex and Hyponex + NAA media showed elongation and differentiation of shoot primordia (Figure 2g,h). Developmental progression and morphology were nearly identical between Hyponex and Hyponex + NAA, indicating that the addition of 1 µM NAA did not significantly enhance germination or early protocorm development. In contrast, SGM-grown protocorms exhibited little change compared with week six, with some showing signs of chlorosis and growth stagnation (Figure 2i).

These findings highlight the critical importance of basal medium composition in the germination and protocorm development of *G. fuscopunctatus*. Orchid seed germination requirements are highly species-specific, and the present results suggest that Hyponex medium is particularly well-suited for supporting the early growth of this species. Similar observations have been reported for other orchids as well. For example, *Dendrobium* cultured on Hyponex medium [23], *Paphiopedilum* on Hyponex medium [9], and *Sedirea* on half-strength Hyponex medium [24] all exhibited markedly improved germination rates and developmental progression, underscoring that the choice of basal medium plays a decisive role in orchid germination and early development.

Furthermore, the lack of a significant effect of NAA supplementation at this stage is consistent with previous reports indicating that the growth-regulating effects of auxins or cytokinins in orchids become more pronounced during later developmental phases, particularly in shoot and root differentiation [25,26]. For *G. fuscopunctatus*, therefore, optimization of the basal medium appears to be the most crucial factor for successful early development, whereas the application of plant growth regulators may be more beneficial during subsequent stages of growth and differentiation.

In summary, this study demonstrates that Hyponex medium is highly effective for seed germination and early protocorm growth of *G. fuscopunctatus*, and that NAA supplementation is not essential at this phase. These findings underscore the importance of basal medium selection in the in vitro conservation of endangered orchids and provide a solid foundation for developing efficient mass-propagation and restoration strategies for this species.

### 2.2. Effect of Different Basal Media on Seedling Growth

The in vitro growth and development of orchid seedlings are strongly affected by the nutrient balance and ionic composition of the basal medium. Over the past decades, several formulations such as Knudson C [27], Vacin and Went [28], Hyponex [29], and Murashige and Skoog [30] have been routinely employed for orchid germination and seedling culture. These formulations provide mineral nutrients essential for protocorm enlargement, chlorophyll synthesis, and subsequent seedling development, but their performance is often highly species-specific and stage-dependent [23,24]. Identifying the most suitable basal medium is therefore critical for successful in vitro propagation and conservation of endangered orchids.

**Figure 3:**
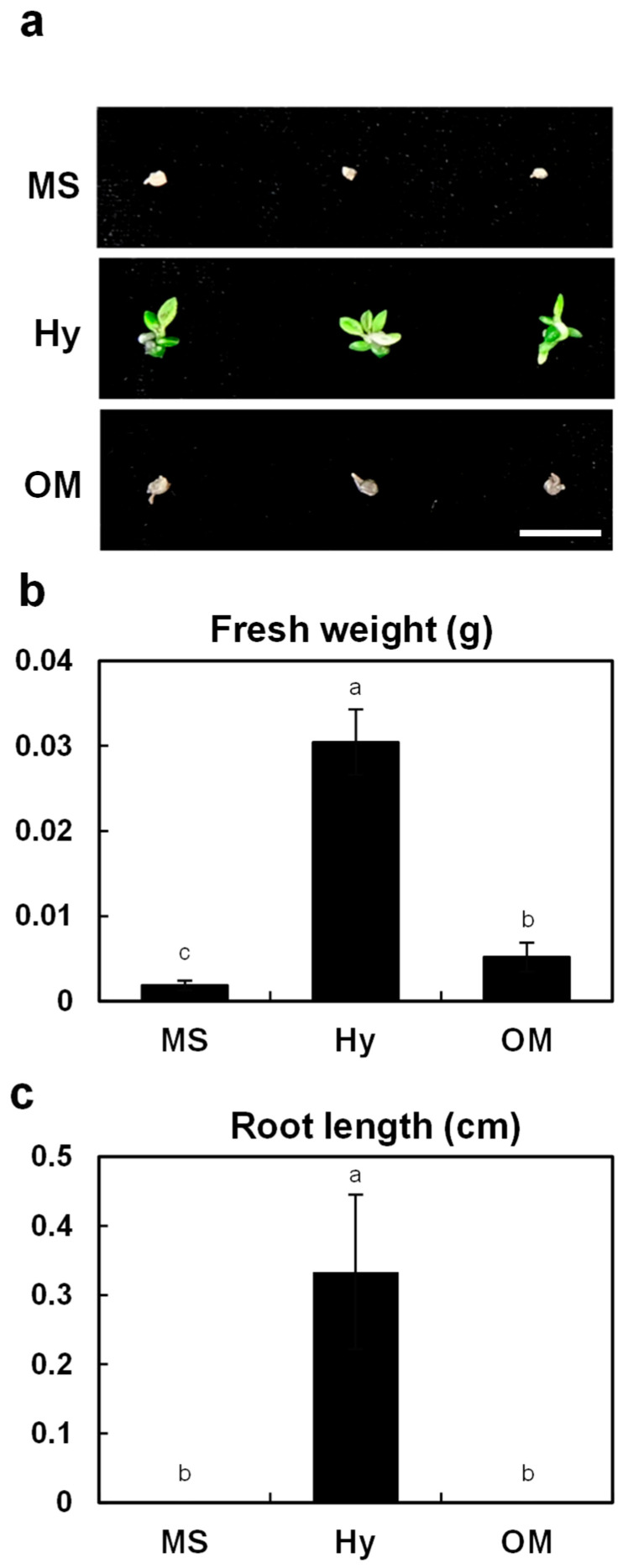
Early seedling growth of *G. fuscopuncta* on different basal media. Growth was evaluated 7 months after sowing. (**a**) Representative seedlings cultured on MS, Hy, and OM media. (**b**) Fresh weight of seedlings. (**c**) Root length of seedlings. Scale bar = 1 cm. MS, Murashige and Skoog; Hy, Hyponex; OM, Orchid Maintenance Medium. Data are presented as means ± SDs from triplicate experiments (*n* = 3). Different letters above the bars indicate statistically significant differences among treatments based on Tukey’s Honestly Significant Difference (HSD) test (*p* ≤ 0.05).

In this study, seedlings of *G. fuscopunctatus* were maintained for seven months on three basal media—MS, Hy, and OM—to evaluate their growth responses. Clear differences were observed among the treatments (Figure 3). Seedlings cultured on Hyponex (Hy) accumulated the greatest biomass, with a fresh weight of 0.0304 ± 0.0038 g, which was approximately 16-fold higher than those on MS (0.0019 ± 0.0005 g) and six-fold greater than those on OM (0.0052 ± 0.0017 g). Root elongation occurred exclusively in Hy (0.33 ± 0.11 cm), while no root development was detected in MS or OM. These results indicate that Hyponex medium provides a nutrient environment particularly favorable for root initiation and overall seedling growth.

Comparable results have been reported in other orchid taxa. An et al. [24] reported that *Sedirea japonica* showed vigorous seedling growth on Hyponex, whereas Hwang et al. [23] confirmed its superiority for *Dendrobium moniliforme*. Conversely, although MS medium remains the standard for plant tissue culture, its relatively high salt strength has often been cited as inhibitory for orchid seedlings unless supplemented with organic additives [31,32]. The findings of Kang et al. [21] further illustrate that while Hyponex was used at the germination stage for *G. fuscopunctatus*, seedlings were transferred to half-strength MS with growth regulators, leaving its seedling-stage potential untested. In contrast, our study demonstrates that continuous culture on Hyponex directly supports robust seedling establishment without the need for supplemental regulators.

The superior growth observed on Hyponex may be explained by its moderate nitrogen content in both ammonium and nitrate forms, which likely promotes balanced assimilation and reduces ionic stress. Such a profile facilitates root initiation and biomass accumulation, in agreement with previous studies on terrestrial orchids such as *Calopogon tuberosus* and *Habenaria macroceratitis* [13,14]. Overall, Hyponex medium proved to be the most effective formulation for *G. fuscopunctatus* seedlings. Its demonstrated ability to enhance biomass production and promote exclusive root development underscores its potential application in ex situ conservation, large-scale propagation, and ultimately, the restoration of endangered orchid populations.

### 2.3. Effect of Organic Additives on Early Seedling Growth

**Figure 4:**
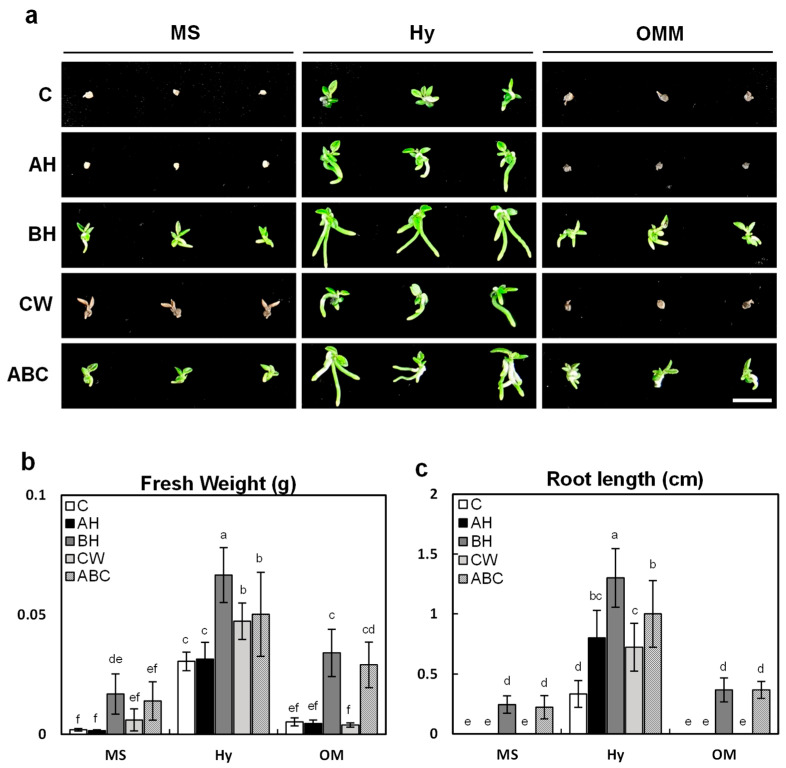
Early seedling growth of *G. fuscopuncta* on different culture media supplemented with organic additives. Growth was evaluated 7 months after sowing. (**a**) Representative seedlings cultured on MS, Hy, and OM media with different organic additives (C, AH, BH, CW, and ABC). (**b**) Fresh weight of seedlings. (**c**) Root length of seedlings. Scale bar = 1 cm. MS, Murashige and Skoog; Hy, Hyponex; OM, Orchid Maintenance Medium; C, control (no additives); AH, apple homogenate; BH, banana homogenate; CW, coconut water; ABC, mixture of all three additives. Data are presented as means ± SDs from triplicate experiments (*n* = 3). Different letters above the bars indicate statistically significant differences among all 15 treatment combinations based on Tukey’s Honestly Significant Difference (HSD) test (*p* ≤ 0.05).

Seven months after sowing, the early growth of *G. fuscopunctatus* seedlings varied markedly depending on the combinations of basal media and organic additives (Figure 4). The overall pattern of growth indicated that the basal medium itself provided the fundamental framework for nutrient uptake, while the type of organic additive determined the degree of enhancement in both biomass and root development. Among the three basal media tested, Hyponex medium consistently supported the most vigorous early seedling development, as reflected in fresh weight accumulation and the initiation of root elongation. In particular, the supplementation of Hyponex with banana homogenate (BH) produced striking improvements, yielding the highest biomass accumulation (0.066 ± 0.01 g fresh weight) and the greatest root elongation (1.3 ± 0.24 cm). These values were substantially greater than those obtained in any other treatment, suggesting a strong synergistic interaction between Hyponex nutrients and the organic compounds provided by BH.

The promotive effect of BH supplementation is consistent with previous findings showing that banana extracts effectively stimulate protocorm differentiation and seedling vigor in diverse orchids. For example, BH significantly enhanced biomass production in *Paphiopedilum primulinum* and *P. glaucophyllum* [33], and concentration-dependent responses were demonstrated in *Serapias vomeracea* [34]. Similarly, Arum & Semiarti [35] reported that BH combined with optimized light treatment markedly improved seedling quality in *Phalaenopsis amabilis*. Although BH supplementation also improved growth on Murashige and Skoog (MS) medium (0.017 ± 0.008 g) and Orchid Maintenance Medium (OM; 0.034 ± 0.009 g), the effect was weaker than in Hyponex, further underscoring the importance of the basal nutrient environment in determining additive efficacy.

Other organic additives, including apple homogenate (AH) and coconut water (CW), generally promoted seedling growth compared with their respective controls. However, their effects were modest, variable, and inconsistent across basal media. This pattern mirrors earlier observations by Momtaj & Kaur [36], who emphasized that organic additives such as BH, CW, and apple juice can stimulate orchid seedling growth but typically exhibit species-specific and concentration-dependent outcomes. Notably, the combined treatment (ABC) did not outperform BH alone, indicating that BH provides the most effective nutritional composition for this species without requiring additional supplements.

The superior performance of BH can be attributed to its content of soluble carbohydrates, vitamins, minerals, and naturally occurring plant growth regulators, including cytokinins and auxin-like compounds [37]. These compounds are likely to stimulate protocorm differentiation, enhance chlorophyll biosynthesis, promote photosynthetic competence, and facilitate efficient nutrient assimilation. Similar mechanisms have been suggested in other orchids such as *Renanthera* [38], *Dendrobium* [24], *Paphiopedilum* [11], and *Phalaenopsis* [3].

In contrast, AH and CW failed to consistently improve seedling performance across all basal media, suggesting that their influence is more context-dependent and possibly concentration-sensitive. Comparable results have been reported in other orchid taxa, where CW stimulated elongation in certain species but showed little effect in others [24,39]. Taken together, the present findings demonstrate that Hyponex supplemented with BH is the most effective medium combination for promoting biomass accumulation and root elongation during the early seedling stage of *G. fuscopunctatus*. While BH supplementation can provide limited benefits when combined with MS or OM, the Hy + BH treatment consistently delivered superior results. However, the efficiency of organic supplements may vary depending on the quality and specificity of the extracts. Therefore, future research and applications should carefully consider the source and preparation methods of the supplements, with particular emphasis on determining the optimal concentration of BH and identifying its key bioactive components to quantitatively assess and refine its role in orchid seedling development.

### 2.4. Effect of Basal Media and Organic Additives on Plant Growth at Later Plant Development Stages

**Figure 5:**
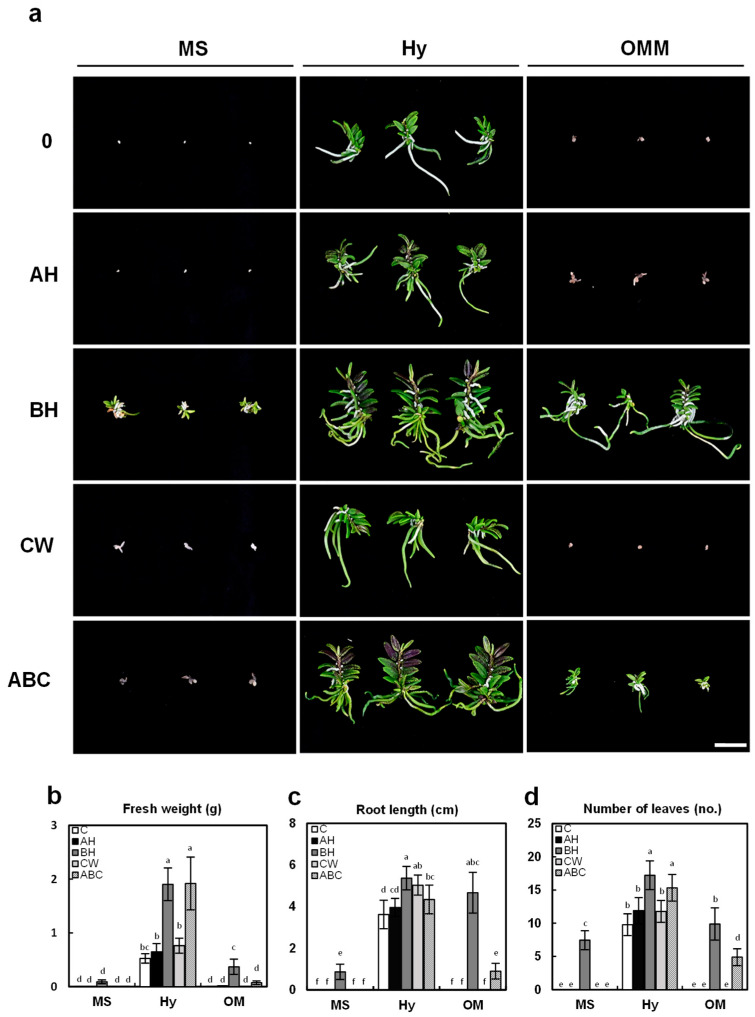
Later-stage plant growth of *G. fuscopuncta* on different culture media supplemented with organic additives. Growth was evaluated 14 months after sowing. (**a**) Representative plants cultured on MS, Hy, and OM media with different organic additives (C, AH, BH, CW, and ABC). (**b**) Fresh weight of plants. (**c**) Root length of plants. (**d**) Number of leaves per plant. Scale bar = 2 cm. MS, Murashige and Skoog; Hy, Hyponex; OM, Orchid Maintenance Medium; C, control (no additives); AH, apple homogenate; BH, banana homogenate; CW, coconut water; ABC, mixture of all three additives. Data are presented as means ± SDs from triplicate experiments (*n* = 3). Different letters above the bars indicate statistically significant differences among all 15 treatment combinations based on Tukey’s Honestly Significant Difference (HSD) test (*p* ≤ 0.05).

At 14 months after sowing, the growth performance of *G. fuscopunctatus* plants showed pronounced differences depending on the basal medium and organic additive combinations (Figure 5). Among the three basal media, Hyponex medium consistently supported the highest biomass accumulation, root elongation, and leaf production. In particular, plants grown on Hyponex supplemented with banana homogenate (BH) exhibited the greatest fresh weight (1.93 ± 0.28 g), root length (5.3 ± 0.56 cm), and leaf number (17.2 ± 2.1), which were significantly higher than all other treatments.

The sustained superiority of BH treatment beyond the early seedling stage supports its role not only in protocorm differentiation but also in long-term vegetative growth. This observation is consistent with BH-mediated growth enhancement reported in *Paphiopedilum* [33] and with the successful reintroduction of *Renanthera imschootiana* through optimized medium composition [38].

The combination of Hyponex with the mixed organic additives (ABC) also performed well, with slightly lower values (1.91 ± 0.49 g fresh weight, 4.34 ± 0.68 cm root length, and 15.3 ± 2 leaves), indicating that BH alone is particularly effective for this species. In contrast, plants grown on MS medium displayed much lower growth parameters, with fresh weight not exceeding 0.1 g in any additive treatment. Similarly, OM medium supported only moderate growth, even when supplemented with BH (0.37 ± 0.14 g fresh weight, 4.7 ± 0.98 cm root length, 9.9 ± 2.4 leaves). These results reaffirm that the basal nutrient composition plays a decisive role in determining growth outcomes, and the balanced macronutrient profile and higher potassium concentration of Hyponex appear to be especially conducive to sustained growth in this species [11].

The superior effect of BH treatment was sustained beyond the early seedling stage (7 months, Figure 4) into prolonged vegetative growth, indicating that BH contributes not only to protocorm development but also to long-term vegetative growth. In contrast, apple homogenate (AH) and coconut water (CW) did not produce consistent or significant improvements in plant growth across any basal medium at the later developmental stage. This trend was consistent with the results observed during the early seedling phase, reinforcing that these additives have limited applicability for this species. Similar concentration-dependent or inconsistent effects of coconut water were also reported in *Dendrobium* and other orchids [23,36]. Furthermore, the sustained lack of response suggests that AH and CW are not suitable for supporting the prolonged vegetative growth of *G. fuscopunctatus*, highlighting the species-specific nature of responses to organic additives reported in orchids.

In addition, recent studies have highlighted that the optimization of basal media and organic additives remains one of the most critical factors for successful orchid conservation. For example, Wongsa et al. [40] demonstrated that basal medium formulation and the presence of specific plant growth regulators decisively affected asymbiotic germination efficiency in endangered orchids. Similarly, Lee et al. [41] reported that coconut water and peptone significantly enhanced protocorm enlargement and chlorophyll content in *Paphiopedilum*, while Fernández et al. [42] confirmed that medium strength and activated charcoal supplementation strongly influenced seedling growth in miniature orchids. These findings, in line with our study, underscore that careful selection and optimization of medium composition and organic supplements are central to developing effective and reproducible protocols for orchid conservation.

One limitation of this study was the relatively small sample size, which was constrained by the endangered status of *G. fuscopunctatus*. Large-scale collection of seeds or seedlings was not feasible due to conservation concerns. Although this reduced the number of replicates, the design was consistent with previous studies on rare orchids, where similar limitations are common. Importantly, the statistical analyses revealed clear and significant treatment effects, indicating that the design was sufficient to detect meaningful biological trends. Future studies utilizing ex situ–propagated material or larger sample sizes will be valuable to further validate and expand upon these findings.

From a conservation perspective, the ability to produce robust, high-biomass plants with well-developed root systems and abundant leaves within 15 months has important implications for the ex situ conservation and reintroduction of *G. fuscopunctatus*. Establishing effective culture protocols not only facilitates propagation of this endangered species but also contributes to broader efforts aimed at the restoration and long-term survival of vulnerable orchid taxa.

## 3. Materials and Methods

### 3.1. Plant Material and Surface Sterilization of Capsules

In December 2023, mature seed capsules of *G. fuscopunctatus* were collected from naturally growing plants in Namhae County, Gyeongsangnam-do, Korea (Figure 1). The capsules were first washed in a neutral detergent solution to remove surface contaminants, followed by three rinses under running tap water to eliminate detergent residues. For surface sterilization, the capsules were transferred to a clean bench and immersed in 70% ethanol for 3 min, then rinsed three times with sterile distilled water to remove any remaining ethanol. They were subsequently soaked in 12% sodium hypochlorite (NaOCl) solution for 15 min with continuous agitation, followed by five rinses in sterile distilled water to completely remove residual NaOCl. Under aseptic conditions, the sterilized capsules were cut longitudinally using a sterile scalpel, and the capsule halves were gently tapped so that the seeds were directly and evenly scattered onto the surface of 100 mL of culture medium in deep square culture vessels (SPL Life Sciences, Seongnam, Korea). All operations were performed under strict aseptic conditions to prevent contamination. Germination was carried out in a growth chamber maintained at 24 ± 2 °C under a 16/8 h light/dark cycle, using cool-white fluorescent lamps at a light intensity of 120 μE m^−2^ s^−1^.

### 3.2. Germination of the Seeds and Protocorm Development

The effects of basal medium type and plant growth regulators on seed germination and protocorm development of *G. fuscopunctatus* were evaluated using three germination media: Orchid Seed Sowing Medium (OSM; P723, PhytoTechnology, Lenexa, KS, USA), Hyponex medium (Kisan Bio, Seoul, Republic of Korea), and Hyponex medium supplemented with 1 μM NAA. The OSM medium was supplied with 1 μM NAA as part of its standard formulation; therefore, no additional NAA was added. All germination media were supplemented with 20 g·L^−1^ sucrose, 1% activated charcoal, and 0.8% (*w*/*v*) agar. The pH was adjusted to 5.6 with 0.1 N KOH prior to autoclaving at 121 °C for 15 min.

Seeds were evenly scattered onto the surface of the three media, and the germination percentage, as well as protocorm development, were monitored under a microscope. Seed germination and protocorm development were classified into seven stages (0–6) as described by Park et al. [20]. Stage 1 was defined as embryo swelling, followed by seed coat rupture, and subsequent shoot and root development. Germination was considered to have occurred when the embryo ruptured through the seed coat. Observations were made at 3, 6, and 8 weeks after sowing. From stage 6 onward, cultures were transferred from dark incubation to light conditions.

### 3.3. Culture Media for In Vitro Seedling Growth

For the assessment of basal medium effects on the growth of *G. fuscopunctatus* seedlings, fifteen protocorms of uniform size and developmental stage (2 months after sowing) were transferred to one of three media types: (1) Murashige and Skoog (MS) medium containing vitamins (Duchefa, Haarlem, The Netherlands), (2) Hyponex medium (N:P:K = 7:6:19; 3 g·L^−1^; Kisan Bio, Seoul, Republic of Korea), and (3) Orchid Maintenance Medium (OMM; PhytoTechnology, Lenexa, KS, USA; P668).

To determine the influence of organic supplements on plants development, protocorms at the same growth stage were cultured on the three basal media with the addition of apple homogenate (AH; 10 g·L^−1^; Kisan Bio, Seoul, Republic of Korea), banana homogenate (BH; 30 g·L^−1^; Kisan Bio, Seoul, Republic of Korea), coconut water (CW; 50 mL·L^−1^; Kisan Bio, Seoul, Republic of Korea), or a mixture containing all three supplements (ABC). The experimental media were as follows: (1) MS + AH, (2) MS + BH, (3) MS + CW, (4) MS + AH + BH + CW, (5) Hyponex + AH, (6) Hyponex + BH, (7) Hyponex + CW, (8) Hyponex + AH + BH + CW, (9) OMM + AH, (10) OMM + BH, (11) OMM + CW, and (12) OMM + AH + BH + CW. The corresponding basal media without organic additives served as controls. All culture media contained 20 g·L^−1^ sucrose, 1% activated charcoal, and 0.8% (*w*/*v*) agar. The pH was adjusted to 5.6 before sterilization by autoclaving at 121 °C for 15 min.

Plants were grown in a controlled-environment growth chamber at 24 ± 2 °C under fluorescent light (120 μmol·m^−2^·s^−1^) with a 16 h light/8 h dark photoperiod. Subculturing was performed at two-month intervals. Throughout the culture period, fresh weight and root length of the plants were periodically recorded. All manipulations were conducted aseptically within a clean bench to avoid contamination.

### 3.4. Measurement of Plant Development and Statistical Analysis

Each treatment consisted of three independent replicates, with ten seedlings per replicate. Fresh weight, root length, and root number were recorded at 2-month intervals after transfer; however, only the results obtained at 7 and 14 months are presented in the figures. Data from all 15 treatment combinations (three basal media × five organic supplement treatments) were analyzed using General Linear Model (GLM), and mean separations were performed using Tukey’s Honestly Significant Difference (HSD) test at a significance level of *p* ≤ 0.05. Statistical analyses were conducted using SAS Studio (version 3.8; SAS Institute Inc., Cary, NC, USA).

**Figure 6:**
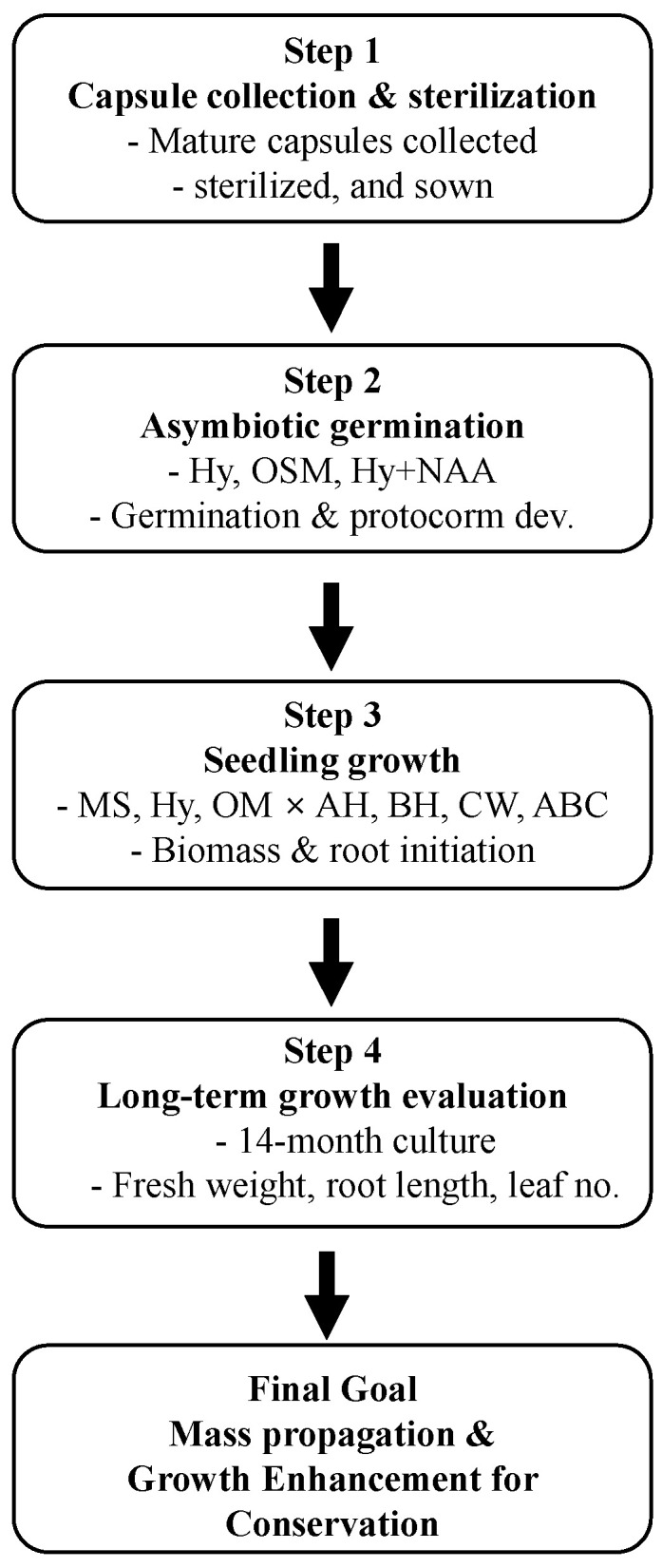
Study map summarizing the experimental workflow for *Gastrochilus fuscopunctatus*. Step 1: Collection and sterilization of mature capsules. Step 2: Asymbiotic germination and early protocorm development on different basal media (Hy, OSM, Hy + NAA). Step 3: Seedling growth evaluation using basal media (MS, Hy, OM) supplemented with organic additives (AH, BH, CW, ABC). Step 4: Long-term growth assessment (14 months), measuring biomass, root length, and leaf number. The final goal of the study was to establish an efficient in vitro propagation protocol to achieve mass propagation and enhanced growth for conservation purposes.

The overall experimental workflow, including seed collection, germination, seedling growth, and long-term evaluation, is summarized in Figure 6.

## 4. Conclusions

This study demonstrated that Hyponex medium supplemented with banana homogenate (BH) provides the most effective environment for both early germination and long-term vegetative growth of *G. fuscopunctatus*. The protocol supported substantial biomass accumulation, well-developed root systems, and abundant leaf formation, offering a reliable strategy for ex situ propagation. The plants produced through this method can be directly applied to habitat restoration and contribute to the recovery of declining wild populations. This underscores the essential role of in vitro culture techniques in the conservation of endangered orchids. Propagation and restoration of *G. fuscopunctatus* also support biodiversity and ecological balance, enhancing ecosystem resilience. In addition to their horticultural value, orchids possess potential pharmaceutical and medicinal applications, further highlighting the importance of sustainable propagation methods. Overall, this study not only established a species-specific propagation system for *G. fuscopunctatus* but also provides practical implications that contribute to global efforts in orchid conservation, ecological restoration, and sustainable utilization.

## Data Availability

The original contributions presented in this study are included in the article material. Further inquiries can be directed to the corresponding author.
